# Catalysts, autocatalysis and the origin of metabolism

**DOI:** 10.1098/rsfs.2019.0072

**Published:** 2019-10-18

**Authors:** Martina Preiner, Joana C. Xavier, Andrey do Nascimento Vieira, Karl Kleinermanns, John F. Allen, William F. Martin

**Affiliations:** 1 Institute for Molecular Evolution, Heinrich-Heine-University, 40225 Düsseldorf, Germany; 2 Institute for Physical Chemistry, Heinrich-Heine-University, 40225 Düsseldorf, Germany; 3 Research Department of Genetics, Evolution and Environment, University College London, Darwin Building, Gower Street, London WC1E 6BT, UK

**Keywords:** hydrothermal vents, catalysis, activation, energy, origin of life, prebiotic chemistry

## Abstract

If life on Earth started out in geochemical environments like hydrothermal vents, then it started out from gasses like CO_2_, N_2_ and H_2_. Anaerobic autotrophs still live from these gasses today, and they still inhabit the Earth's crust. In the search for connections between abiotic processes in ancient geological systems and biotic processes in biological systems, it becomes evident that chemical activation (catalysis) of these gasses and a constant source of energy are key. The H_2_–CO_2_ redox reaction provides a constant source of energy and anabolic inputs, because the equilibrium lies on the side of reduced carbon compounds. Identifying geochemical catalysts that activate these gasses en route to nitrogenous organic compounds and small autocatalytic networks will be an important step towards understanding prebiotic chemistry that operates only on the basis of chemical energy, without input from solar radiation. So, if life arose in the dark depths of hydrothermal vents, then understanding reactions and catalysts that operate under such conditions is crucial for understanding origins.

## 1. Introduction

When the Earth was formed 4.5 billion years ago, it was formed without life, we can safely presume. If there was any life on the freshly accreted Earth, it was destroyed at the moon forming impact, which converted the Earth into a ball of boiling magma [1]. By about 3.95 billion years ago, there was life on Earth [2]. The question of how it arose is of substantial interest. Hydrothermal vents play an important role in the question of life's origin, because they were present on the early Earth [3–7] and because they harbour continuously far-from-equilibrium conditions in an environment where H_2_ and CO_2_ interact in such a way as to generate reduced carbon compounds [8–15]. In the discussion of possible sites for life's origin, hydrothermal vents are unique by that criterion: hydrothermal vents harbour far from equilibrium conditions over geological timescales, and the approach towards equilibrium releases energy in the synthesis of reduced carbon compounds. This sets hydrothermal vents apart from all other physico-chemical settings [16]. Moreover, the release of free energy and the synthesis of reduced carbon compounds at vents are united in a common reaction sequence that operates in the laboratory without enzymes [15] and that is simultaneously the core of carbon and energy metabolism in real bacteria and archaea—acetogens and methanogens. Vents are unique among settings for the origin of *metabolism* (as opposed to the origin of *life*), because no other site for life's origin harbours chemical reactions that resemble real microbial carbon and energy metabolism.

The far-from-equilibrium conditions at alkaline hydrothermal vents entail steep redox gradients owing to a constant flux of H_2_-rich effluent over geological timescales [17]. The main redox reaction they harbour is the H_2_–CO_2_ system, in which the equilibrium lies far on the side of organic compounds [18], such that the reaction can proceed spontaneously as long as suitable catalysts are available and strictly reducing conditions are maintained [10,15,19,20]. In the presence of activated nitrogen species, hydrothermal vents can synthesize the building blocks of life [12,13]. Because of their abundance of chemical energy, and despite the absence of light, modern alkaline hydrothermal vents are teeming with microbial life [21,22], life that is ultimately fuelled by the reaction of H_2_ with CO_2_.

The H_2_–CO_2_ redox reaction is an attractive source of energy for the first chemical reactions en route to life, because it provides direct links between a known geochemical process (serpentinization) and known biochemical processes. These are most notably the reactions of core carbon and energy metabolism in acetogens and methanogens, anaerobic autotrophs that live from the reduction of CO_2_ with H_2_. Acetogenesis and methanogenesis represent the most primordial forms of metabolism in bacteria and archaea [23,24], rooting life's chemistry to reactions of gasses, rocks and water.

The continuity between exergonic geochemical and biochemical reactions can be seen as a virtue of hydrothermal origin theories, because it generates concrete mechanistic links between processes catalysed by minerals in the Earth's crust (exergonic CO_2_ reduction) [25] and processes catalysed by enzymes in the metabolism of prokaryotic lineages [26]. At hydrothermal vents, life as we know it connects to geochemistry as we know it.

## 2. Activation of CO_2_ and H_2_: the door to CO_2_ fixation

In biology, acetogens and methanogens fix CO_2_ via the H_2_-dependent reduction of CO_2_ to a methyl group and CO, followed by condensation of the methyl moiety and CO to a nickel bound acetyl group that is thiolytically cleaved from nickel to generate the thioester acetyl-CoA. The acetyl-CoA pathway is unique in microbial physiology, because it is carbon and energy metabolism in one. Carbon metabolism involves the H_2_-dependent reduction of CO_2_ to acetyl-CoA. Under standard physiological conditions, the synthesis of the thioester is exergonic by about –59 kJ mol^−1^ [27], while there is not enough energy to generate thioesters and synthesize ATP via substrate level phosphorylation [28]. Thus, for energy metabolism, acetogens that lack cytochromes and quinones couple methyl synthesis to the generation of ion gradients via electron bifurcation and ferredoxin oxidation at the membrane-bound Rnf complex [29], while methanogens that lack cytochromes generate their ion gradient by coupling the transfer of the methyl group from a nitrogen atom in methyl-tetrahydromethanopterin to a sulfur atom in coenzyme M [30].

If the acetyl-CoA pathway is the most ancient carbon fixation pathway, and various lines of evidence indicate that to be the case [14,15,23,24,27,31], there are still some dots that need to be connected. For H_2_ to have played a role in early chemical evolution, it required activation—it required catalysis. It is noteworthy that H_2_ never interacts directly with any organic oxidant (substrate) in metabolism, it always releases electrons into metabolism via a catalyst: hydrogenase. There are only three classes of hydrogenases known. All three harbour Fe atoms at their active site [32,33], all three harbour carbon metal bonds at their active site [26]. The central enzyme of the acetyl-CoA pathway, the only exergonic CO_2_ fixation pathway known [34,35], is bifunctional carbon monoxide dehydrogenase/acetyl-CoA synthase (CODH/ACS), which also harbours carbon metal bonds. These two activities, hydrogenase and CODH/ACS, trace to the last universal ancestor, LUCA [26]. Organisms that use the acetyl-CoA pathway employ flavin-based electron bifurcation to generate ferredoxins with a lower reducing potential than H_2_ [36–38]. Flavin-based electron bifurcation thus accounts for the thermodynamics of H_2_ oxidation, but what about the kinetics? In kinetically controlled reactions, catalysts can have an important influence on the nature of the products that accumulate—and the same is true for geochemical CO_2_ fixation with H_2_.

**Figure 1.**
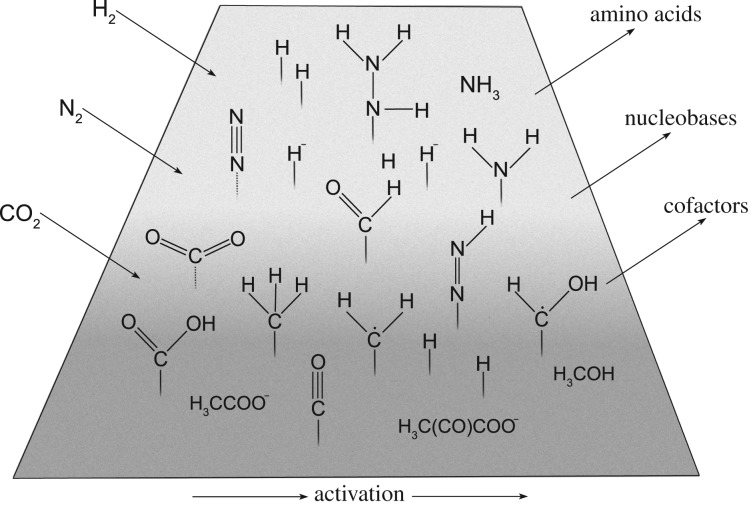
Simultaneous activation of H_2_, CO_2_ and N_2_ on mineral surfaces leading to the formation of a variety of biologically relevant molecules, such as amino acids, nucleic acid bases and cofactors. Molecules, such as pyruvate, acetate, methanol and ammonia, are known to form on transition metal containing surfaces [15,45]. Little is known about the products obtained when the separation of N and C fixation is revoked. Heterogeneous catalysis may have been the key for early processes of protometabolism. Dashed lines indicate physisorption, non-dashed lines indicate chemisorption on the surface.

The H_2_-dependent reaction from the most oxidized form of carbon, CO_2_, to its most reduced form, methane (CH_4_), is thermodynamically favourable under reducing conditions. However, in serpentinizing, alkaline hydrothermal systems [39] the direct transfer of electrons from H_2_ to CO_2_ has a large activation energy and requires either high temperatures and high pressures [40] or, at milder conditions, chemical activation and catalysis [41,42]. The requirement for catalysis stems from kinetic barriers in the sequence of reactions from CO_2_ to CH_4_. Catalysts decrease the activation energy and thus the kinetic barrier, allowing intermediate products such as formate, acetate, methanol and pyruvate to accumulate after a short time under mild conditions [15] rather than the thermodynamically favoured end product CH_4_. While high temperatures, high pressures and long reaction times lead to the accumulation of CH_4_, the most stable product [40,43], catalysts influence the product distribution in the short term. In biology, enzymes effect such shifts from thermodynamically controlled reactions to kinetically controlled reactions [44]. In purely geological settings, however, heterogeneous catalysis can occur on mineral surfaces (figure 1)—which are not unlike the catalysts used in industry to produce hydrocarbons [15,25]. The activation of molecules on mineral surfaces is likely to have preceded the chemical activation that enzymes provide in modern organisms [46,47].

## 3. Adding nitrogen

In order to synthesize amino acids and nucleic acid bases, living cells have to incorporate dinitrogen (N_2_) into biosynthetic pathways. From a chemical point of view, N_2_ as a starting material is not the easiest choice in comparison to more oxidized or reduced nitrogen compounds [48]. Nevertheless, looking at early Earth's conditions, an atmosphere filled with N_2_ would have led to an ocean with dissolved N_2_ and thus—via sequestration through the Earth's crust—to a nitrogen source in serpentinizing systems [49,50]. Looking at biology, N_2_ fixation is considered ancient [50,51]. There is only one way for N_2_ to enter metabolism: via the nitrogenase complex. Nitrogenase consists of two proteins, dinitrogenase reductase, which contains an FeS-based active centre and the dinitrogenase protein, harbouring an Mo (or V, or Fe) containing Fe_7_S_9_ centre with a carbide carbon at the active site [52,53]. Mechanistically, the complex works with dinitrogenase reductase harvesting the energy of ATP hydrolysis and transferring it via conformational changes to dinitrogenase, which then binds the N_2_ molecule [53,54]. The following steps involve sequential hydrogenations of the nitrogen molecule. There, as for CO_2_ fixation, hydrogenase activity is needed to deliver electrons from H_2_ to N_2_. This hydrogenase activity is promoted by the FeS clusters of the nitrogenase complex [53,55], which is the sole entry point of N_2_ into metabolism. As CODH and hydrogenase, nitrogenase also traces back to LUCA [26,56].

Biology operates within constraints of temperature and pressure. Biological N_2_ reduction follows very different kinetics from those of the industrial process [57]. For both processes, inorganic catalysts have a central role in the reduction of N_2_. In industry, the reduction of N_2_ might resemble prebiotic FeS-based nitrogen fixation [45]. The greatest impediment to N_2_ reduction is its activation energy. N_2_ is very stable at normal atmospheric temperatures and pressures. Thus, few processes are capable of activating N_2_ sufficiently in order to form N-rich molecules. Industrial N_2_ conversion to NH_3_ via the Haber–Bosch process (H_2_-dependent) requires Fe-based catalysts such as Fe_3_O_4_, high pressure (200 bar) and temperatures exceeding 400°C [58]. The Haber–Bosch process currently consumes about 1–2% of the World's total energy production. Biological nitrogen fixation catalysed by the nitrogenase enzyme operates at ambient pressure and room temperature. Accordingly, there is immense commercial interest in the mechanism of biological N_2_ fixation [57].

Not unlike the stepwise use of Fe atoms found in the active sites of the nitrogenase complex, industrial N_2_ reduction is extremely dependent on the physico-chemical state of the catalysts. Thus, the yield of ammonia is affected as a result of several factors such as particle size, purity and subsurface dissociation of nitrogen into Fe catalysts, leading to iron nitrides such as Fe*_x_*N [59].

Can serpentinization reduce N_2_? Although there is abundant evidence for abiotic CO_2_ reduction in serpentinizing systems [60,61], evidence for abiotic N_2_ reduction is so far lacking. Laboratory simulations suggest that N_2_ can be reduced to ammonia (NH_3_) with mineral catalysts under mild hydrothermal conditions [45,62]. Incorporation of N from N_2_ into organic compounds under hydrothermal conditions presents a more substantial challenge for laboratory simulations. In principle, activated forms of nitrogen chemisorbed to geochemical catalysts (figure 1) might be better starting points for prebiotic synthesis of such compounds than NH_3_ [25], but this remains to be shown experimentally.

There are nevertheless very curious parallels between industrial hydrogenation processes and geochemical H_2_-dependent reactions. Serpentinization not only reduces H_2_O to H_2_ and CO_2_ to formate and CH_4_, it also generates inorganic catalysts within the Earth's crust [25]. These include magnetite, Fe_3_O_4_, which is the catalyst of choice for the industrial Haber–Bosch process (H_2_-dependent N_2_ reduction) and for Fischer–Tropsch (CO_2_ reduction) applications [59,63] and awaruite, Ni_3_Fe, which catalyses the H_2_-dependent reduction of CO_2_ to methane at high pressures and temperatures [40]. While H_2_ and CO_2_ deliver carbon and energy, for an autocatalytic network to emerge, one from which microbial metabolism could unfold, organic cofactors, bases and amino acids are required. All are nitrogenous compounds.

## 4. What if C, N and H are activated together?

As shown in figure 1, it is possible that mineral surfaces can activate H_2_, CO_2_ and N_2_ simultaneously. If so, amino acids or even bases and cofactors might be obtained via such routes. It has been reported that Fe^2+^ and Fe^0^ can catalyse reactions of 2-oxoacids with hydroxylamine to give aspartate, alanine, glycine and glutamine [64]. These should also be the first amino acids to appear in the evolution of metabolism, if metabolism evolved from a pyruvate-fed, incomplete citric acid cycle and if amino acids arose ancestrally as they do in metabolism, namely via reductive amination of the keto group in oxalacetate, pyruvate, glyoxylate and 2-oxoglutarate [9]. Pyruvate is new as a possible prebiotic compound [14]. Using hydrothermal iron minerals instead of enzymes, it is possible to synthesize pyruvate from H_2_ and CO_2_ [15]. Pyruvate now appears to be a much more readily synthesized prebiotic compound than previously assumed.

**Figure 2.**
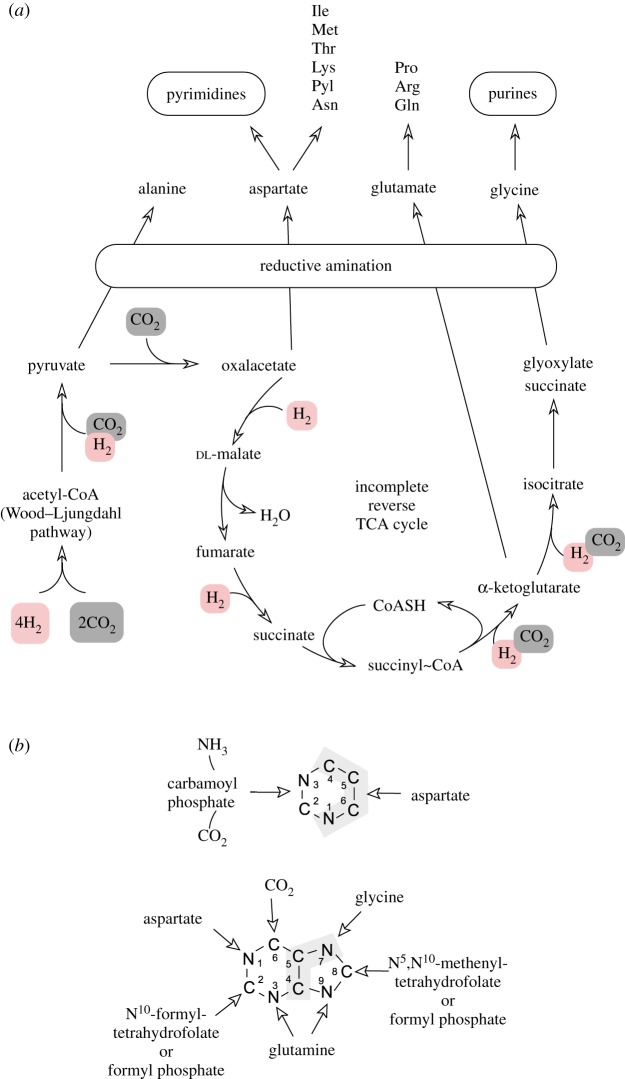
A path from H_2_ and CO_2_ to nucleic acid bases (adapted from figs. 3, 4 and 6 of [9]. (*a*) The lower portion of the panel shows the biosynthesis of carbon backbones in microbes that use the acetyl-CoA pathway and the incomplete (horseshoe) reverse citric acid cycle. Reductive steps of CO_2_ fixation are indicated as H_2_-dependent, though reduced ferredoxin or NAD(P)H are the reductants in metabolism. The first four 2-oxoacids to arise via the route shown, and, if reductively aminated, generate Ala, Asp, Glu and Gly (upper portion of the panel). Muchowska *et al*. [64] showed that pyruvate, oxaloacetate, 2-oxoglutarate and glyoxylate are readily reduced to the corresponding amino acids by hydroxylamine under mild conditions in the presence of native iron. Asp is the starting point for biosynthesis of five other canonical amino acids and pyrrolysine, Glu is the starting point for synthesis of Gln, Arg and Pro. (*b*) Asp and Gly are central to pyrimidine and purine biosynthesis, respectively (modified from fig. 4 of [9]). The involvement of CO_2_ in purine and pyrimidine synthesis is noteworthy, as is the involvement of folate-bound C1 intermediates of the acetyl-CoA pathway in purine synthesis, which are replaced by the simpler intermediate formyl phosphate in methanogens. This suggests the possibility of a small prebiotic biochemical network linking CO_2_ reduction to nucleic acid base synthesis. (Online version in colour.)

If N_2_ can be activated efficiently under hydrothermal conditions, nucleic acid bases might not be far away. Recent studies show that even aromatic heterocyclic compounds such as tryptophan can be formed abiotically in serpentinizing hydrothermal systems [13]. The connection of simpler amino acids like aspartate and glycine to bases is direct, they sit in the middle of the aromatic pyrimidine (aspartate and glycine) and purine (aspartate) rings. This is shown in figure 2, modified from reference [9]. In metabolism, pyrimidines are made from aspartate and carbamoyl phosphate. Carbamoyl phosphate is made from carbamate and ATP, carbamate forms spontaneously as a colourless precipitate in hot solutions containing CO_2_ (or carbonate) and ammonium. Four of the atoms in the pyrimidine ring come from aspartate. Purines are more complex, but the components are simple. Glycine comprises the centre of the rings, which are completed by inclusion of C1 units from formyl tetrahydrofolate [65] or from formyl phosphate (in methanogens) [66], by N from the amido group of glutamine, and, as with pyrimidines, by CO_2_ and N from aspartate.

There is a clear record of geochemical origins preserved in metabolism [26]. This record can be resurrected in the laboratory, if we find the right conditions. The four amino acids that Muchowska *et al*. [64] synthesize (Gly, Ala, Asp, Glu) even suggest (reveal, one might say) a connection to the evolution of the genetic code. These are the very same amino acids that are identified as ancient in different theories about the origin and evolution of the genetic code. In some theories, exactly these four (Gly, Ala, Asp, Glu) are the oldest [67]. In other theories, they are the most ancient as members of larger sets [68], while in yet other theories they rank well in order of antiquity, with Gly, Ala and Asp being the oldest, Glu coming in seventh [69]. A look at the biosynthetic families of amino acids reveals that the Asp and Glu families stand out as central.

## 5. Autocatalytic networks

If we assume that simultaneous activation of N_2_, H_2_ and CO_2_ can lead to thermodynamically stable products that include amino acids, nucleic acid bases and cofactors (that is currently a big assumption, we admit), then small chemical networks on a laboratory scale become possible. Central to various schools of thought on chemical origins are constructs called autocatalytic networks [70]. These can represent abstract mathematical constructs or they can describe interactions in real sets of molecules. As applied to molecular interactions, autocatalytic networks contain molecules that promote the synthesis of copies of themselves [71]. According to this very general definition, autocatalytic networks can provide theoretical frameworks for both the genetics first and the metabolism first approaches to prebiotic evolution. In the former, they can be sets of nucleic acids that ligate to form specific products [72], in the latter, they can be sets of metabolites that interact in such a way as to generate self-sustaining metabolic networks [24].

When describing molecular interactions, autocatalytic sets require input molecules in order to promote the synthesis of their constituent elements. This condition draws attention to a particular class of autocatalytic networks called reflexively autocatalytic food-generated networks—RAFs [73]—in which each reaction is catalysed by a molecule from within the network, and all molecules can be produced from a set of food molecules by the network itself. RAFs are particularly interesting in the context of early evolution, because they do not require a pre-existing catalyst for a reaction before it is required. The reaction can proceed uncatalysed, or rather catalysed by an unknown molecule, as long as the known catalyst is produced at some point by the network and assumes the role of catalysis in that reaction of the RAF. Moreover, when it comes to the concrete modelling of early evolution, the nature and source of the food molecules [74] that generate a given RAF or other autocatalytic set are of particular interest, because in order for the reactions in the set to take place, the overall thermodynamics of the network must be exergonic. In other words, in order for RAFs (or other autocatalytic networks) to serve as a useful model for early evolution, the set of reactants (educts) needs to release energy en route to the products (adducts), as is always the case in metabolism [18].

Of course, in cellular metabolism, the overall energetics are given by the sum of the changes in free energy for the core bioenergetic reactions [18]. For individual reactions of metabolism, the change in free energy from substrate to product is often endergonic, which is why such reactions are usually coupled to energy-releasing reactions involving exergonic electron transfer, ion gradients across the plasma membrane, or hydrolysis of high-energy bonds, such as ATP, acyl phosphates or thioesters [18,37]. Energetic coupling can also occur within RAFs, which makes them more interesting models of cellular metabolism.

It seems likely that at least a subset of the catalysts, high-energy bonds and energetic currencies that occur in modern metabolism were generally present and functional in prebiotic chemistry. Sources and transduction of modern metabolic catalysis and energy should then have analogues or homologues in geochemical settings. Regarding catalysis, there are now good indications that metals and simple organic cofactors could have promoted the emergence of cell-sized autocatalytic networks [15,64,75,76]. In physiology, the term energy metabolism generally means ATP synthesis. There are two sources of ATP in cells: chemiosmotic coupling and substrate level phosphorylation (SLP). Chemiosmotic coupling needs ion gradients as an energetic intermediate and proteins, without exception. SLP does not require ion gradients, its energy source is the Gibbs free energy of chemical reactions, and SLP reactions can take place without enzymes [77–79]. Although vents harbour natural ion gradients, ATP synthesis via chemiosmotic coupling always involves the ATPase, for which there is no known geochemical homologue or mechanistic analogue. The energy for SLP stems from the redox chemistry of carbon whereby both carbon oxidation to CO_2_ and H_2_-dependent CO_2_ reduction can be coupled to SLP [80]. Because the H_2_-dependent CO_2_ reducing reaction that drives SLP in acetogens (acetate synthesis) operates in the laboratory under simulated hydrothermal vent conditions with only metals and metal ions as catalysts [15], it is currently the only candidate for a primordial (geochemical) source of energy conservation (acyl phosphates via SLP) that is mechanistically linked to naturally occurring carbon redox reactions at vents.

**Figure 3.**
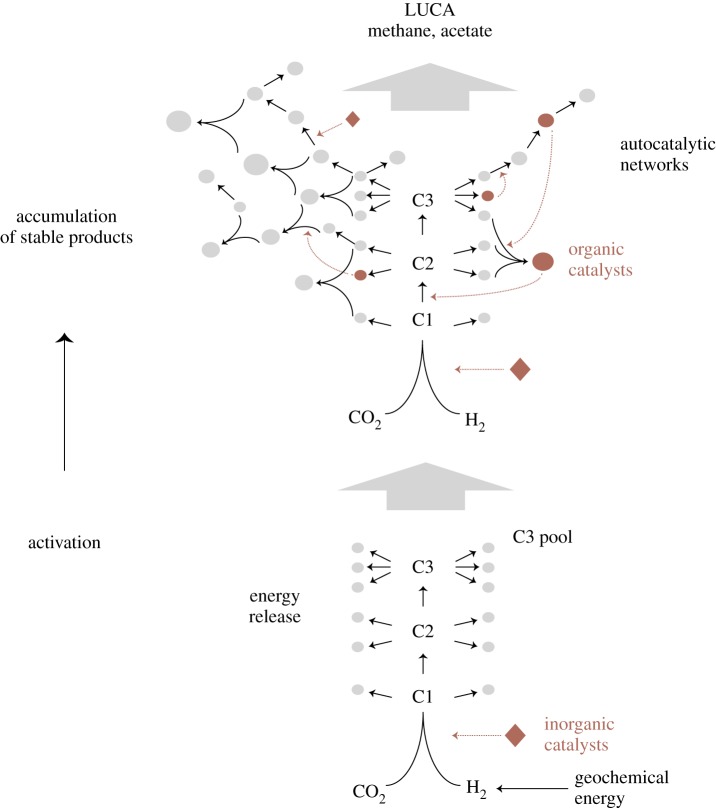
Purely geochemical reactions such as CO_2_ fixation with H_2_ can give rise to autocatalytic networks and protometabolism, as long as energy is released. Kinetically controlled reactions build up a specific set of products which interact further to form an autocatalytic network that serves as a basis for higher complexity. C1, C2, C3 represent carbon compounds with 1, 2 or 3 carbon atoms such as formyl groups, acetyl groups, and pyruvate. (Online version in colour.)

A set of molecules that is generated by kinetically controlled reactions (the most rapidly formed products accumulate) will contain chemical energy that permits members of the set to interact further and to form an autocatalytic network that can serve as a basis for higher complexity [76]. Such a process is sketched in figure 3. The energetic input is necessarily centralized because thermodynamically stable metabolites and end products are synthesized from the core exergonic reaction, in our example the reduction of CO_2_ with H_2_ via the acetyl-CoA pathway [9,15,31].

## 6. Conclusion

Hydrothermal vents contain catalysts and chemical disequilibria that resemble life and metabolism in many ways. However, the natural chemical environment at vents does not strongly resemble metabolism in many forms of life, because metabolism is extremely diverse. Rather, it very specifically resembles the physiology of acetogens and methanogens, even down to the catalysts involved. The connections between the origin of microbial life and the chemical elements seem more tangible than ever before. Current genomic analyses indicate that the last universal common ancestor of all life, LUCA, lived from gasses: H_2_, CO_2_ and N_2_ [23,56]. Although our main focus is on these three gasses, it is evident that the incorporation of sulfur (S) and phosphorus (P) into early metabolism was also essential. Sulfur enters metabolism as HS^–^ at cysteine synthesis from *O*-acetyl serine or *O*-phospho serine [81], while phosphorus enters metabolism via thioesters as acyl phosphates [9]. Under reducing conditions, H_2_S (HS^–^ in alkaline vents) would be the likely sulfur source, phosphorus could enter the geochemical setting as phosphate dissolved in seawater or leached from the primordial crust, but data on phosphate under early Earth conditions is scarce [82–84]. Focusing on the enzymes that channel H_2_, CO_2_ and N_2_ into metabolism might uncover clues about the environment within which life arose and about the catalysts that activated these gasses at origins. The presence of carbon metal bonds in the active sites of hydrogenases, nitrogenase and carbon monoxide dehydrogenase suggest that these might be ancient relicts of the catalytic realm that led to the autocatalytic synthesis of the first organic compounds. We propose that the biology of methanogens and acetogens, anaerobic autotrophs that inhabit vents today, holds clues about the primordial catalysts that enzymes ultimately came to replace.

## Data accessibility

This article has no additional data.
